# Oral Doxycycline Reduces Pterygium Lesions; Results from a Double Blind, Randomized, Placebo Controlled Clinical Trial

**DOI:** 10.1371/journal.pone.0052696

**Published:** 2012-12-19

**Authors:** Oscar Rúa, Ignacio M. Larráyoz, María T. Barajas, Sara Velilla, Alfredo Martínez

**Affiliations:** 1 Ophthalmology Service, Hospital San Pedro, Logroño, Spain; 2 Oncology Area, Center for Biomedical Research of La Rioja (CIBIR), Logroño, Spain; 3 Pharmacy, Hospital San Pedro, Logroño, Spain; Univeristy of Melbourne, Australia

## Abstract

**Purpose:**

To determine whether oral doxycycline treatment reduces pterygium lesions.

**Design:**

Double blind, randomized, placebo controlled clinical trial.

**Participants:**

98 adult patients with primary pterygium.

**Methods:**

Patients were randomly assigned to receive 100 mg oral doxycycline twice a day (49 subjects), or placebo (49 subjects), for 30 days. Photographs of the lesion were taken at the time of recruitment and at the end of the treatment. Follow-up sessions were performed 6 and 12 months post-treatment. Statistical analyses for both continuous and categorical variables were applied. *p* values of less than 0.05 were considered to indicate statistical significance.

**Main Outcome Measures:**

The primary endpoint was the change in lesion size after 30 days of treatment.

**Results:**

The primary endpoint was not met for the whole population but subgroup analysis showed that doxycycline was effective in patients of Caucasian origin while other ethnicities, mostly Hispanic, did not respond to the treatment. Moreover, there was a correlation between age and better response (*p* = 0.003). Adverse events were uncommon, mild, and in agreement with previous reports on short doxycycline treatments.

**Conclusions:**

Oral doxycycline was superior to placebo for the treatment of primary pterygia in older Caucasian patients. These findings support the use of doxycycline for pterygium treatment in particular populations.

**Trial Registration:**

European Union Clinical Trials Register EudraCT 2008-007178-39

## Introduction

Pterygia are common, benign, tumor-like growths of the cornea [1]–[3]. The condition is characterized by an advancing edge of epithelial cell overgrowth with squamous metaplasia, goblet cell hyperplasia, and abnormal p53 expression [4], [5]. The underlying stroma presents abundant fibrosis and rampant angiogenesis [6], [7]. Without surgical intervention, these characteristically wing-shaped lesions can migrate over the central cornea and distort or obscure vision. Human pterygia express, among other factors, fibronectin, which is associated with cell adhesion and migration, as well as pro-inflammatory cytokines, angiogenic, and fibrogenic growth factors, such as vascular endothelial growth factor (VEGF), transforming growth factor β (TGFβ), interleukin 6, interleukin 8, versican, and CD31 antigen [2], [8]–[10]. The relevance of angiogenesis in primary and recurrent pterygium has been recently used to conduct clinical trials with topical treatments using anti-VEGF agents [11]–[14]. On the other hand, no systemic treatment has been developed for this disease.

Doxycycline is an affordable bacteriostatic antibiotic that has been used safely for decades in the clinic. It has been shown that, independently of its antimicrobial effect, doxycycline can suppress the catalytic activity of many matrix metalloproteinases (MMP), including gelatinases and collagenases, and through this mechanism reduce cell migration and angiogenesis [15]. In a study using human endothelial cells, doxycycline was able to inhibit MMP activity, protein synthesis, and mRNA expression [16]. Doxycycline has also been used in several animal models of neovascularisation or tumorogenesis with very promising results [17]–[20]. Recently, we developed a mouse model of pterygium and demonstrated that oral doxycycline drastically reduced pterygium lesions in this experimental model [21]. In addition, treatment of short-term pterygium cultures with doxycycline for 24 h resulted in a sharp change in expression of mitochondrial genes, activation of the endoplasmic reticulum stress response and profound changes in the expression of extracellular matrix proteins, adhesion molecules, and growth factors [22]. The very affordable economic value of this drug together with its safety profile made doxycycline a promising therapeutic candidate for the treatment of pterygium and other ocular diseases.

Here we present the results of a double blind, randomized, placebo controlled clinical trial in patients suffering from primary pterygium comparing 30 days of treatment with oral doxycycline vs. placebo. Results were very influenced by the race and age of the patients, with Caucasian and older people benefiting most from the treatment.

## Materials and Methods

### Ethical issues

All procedures were approved by the local review board (Comité Ético de Investigación Clínica de La Rioja, CEICLAR) and the Spanish Agency for Drugs and Medical Products (Agencia Española de Medicamentos y Productos Sanitarios, AEMPS). All described procedures adhere to the tenets of the Declaration of Helsinki. Moreover, the trial was registered in the European Union Clinical Trials Register with code number EudraCT 2008-007178-39. The protocol for this trial and supporting CONSORT checklist are available as supporting information; see Checklist S1, Protocol S1 (in Spanish), and Protocol S2 (in English).

### Patients

The study was designed as a unicentric trial with patients diagnosed with primary pterygium at the Ophthalmology Service of the Hospital San Pedro (Logroño, Spain). Ninety-eight consecutive patients fulfilling inclusion criteria signed the informed consent documents and were recruited into the trial. Inclusion criteria called for patients suffering from untreated primary pterygium producing at least one of the following symptoms: i) astigmatism with no other cause, ii) foreign body sensation, and/or iii) corneal involvement threatening the visual axis. In addition, patients had to be at least 18 years old. Exclusion criteria encompassed pregnant or lactating women, fertile women not following a contraceptive plan, allergies to doxycycline, conditions where doxycycline is contraindicated such as lupus eritematosus or miastemia gravis, treatment with doxycycline incompatible drugs, and patients whose characteristics would prevent proper follow-up.

The number of patients needed on each arm of the study was estimated assuming a mean reduction of the pterygium lesion in the placebo group of 5% vs. a 15% reduction for the doxycycline group, and a maximum standard deviation of 15%. Using a Student's *t* test for 2 independent samples, a two-sided alpha level of 5%, and a power of 90%, 49 patients are needed on each arm to detect a significant difference between groups of 10% in pterygium surface reduction.

### Experimental drug and placebo

Doxycycline was provided as Vibracina© 100 mg (Invicta Farma, Madrid, Spain). Capsules were extracted from their blisters and repackaged by the Hospital's Pharmacy in brown-glass bottles containing 60 capsules/each. The placebo was prepared by the Hospital's Pharmacy using empty Vibracina© capsules, that were generously provided by Invicta Farma. These capsules were filled up with lactose solution, desiccated, closed, and packaged in brown-glass bottles (60 capsules/bottle). Each bottle (doxycycline or placebo) was assigned a randomly generated trial code and issued to patients accordingly, thus guaranteeing a double-blind masking of the trial. The dose of 200 mg/day was chosen based on the study by Smith et al. where they described this dose as the most efficient in reducing MMP activity in patients [23].

### Study design and procedures

Each patient completed 4 visits to the Ophthalmology Department. On the first visit, a diagnosis of the pterygium was made and clinical history data were collected. If the patient fulfilled the inclusion criteria and none of the exclusion criteria applied to him/her, he/she was asked to be included in the trial. After signing the informed consent forms, a code was assigned to this patient for the duration of the trial. To estimate the size of the lesions, the diameter of the cornea was first measured with a compass, then a photograph was taken of the affected eye (or both in the case of bilateral pterygia) with a Zeiss FF 450 plus I.R. camera (Carl Zeiss, Meditec AG, Berlin, Germany) attached to a retinographer. The size of the pterygium lesion lining the cornea was calculated with the camera's image software package (Visupac^TM^, Carl Zeiss), taking into consideration the compass measurement. Pterygia were classified as T1, T2 or T3, according to Tan's grading system [24]. Using his/her code, the patient was given a bottle of capsules by the Hospital's Pharmacy and the pertinent contact information in case adverse events may occur. Patients were asked to take 2 capsules a day, in the morning and the evening, for 30 consecutive days.

The second visit was scheduled 31 days after the first one, just as the patient had finished the treatment. At this time a second photograph was taken of the affected eye(s) and a general evaluation was made. After this point, the ophthalmologists performed surgical resections of the pterygia whenever the procedure was clinically indicated. In these cases, the procedure consisted in a simple resection followed by autologous conjunctival transplant and application of a fibrin-based biological glue (Tissucol, Baxter, Valencia, Spain).

The third and fourth visits occurred 6 and 12 months after the second one. The ophthalmologists performed follow-up observations and paid special care to record potential recurrences.

### Objectives and outcomes

The main objective of this study was to determine whether oral doxycycline treatment can reduce pterygium growth. Thus, the primary outcome was the variation in the surface area occupied by the pterygium lesion when comparing the photographs taken during the second and the first visit. Photographs were processed with Visupac^TM^ and ImageJ (NIH, Bethesda, MD) and the area occupied by the lesion calculated. As secondary outcome, the number of recurrences at the end of the study (4^th^ visit) were also considered.

### Statistical analysis

The variation of surface area occupied by the pterygium lesion, a continuous variable, was compared between the two experimental groups with the Student-Fisher *t* test. Normalcy was determined by the Shapiro-Wilk test. Subgroup analysis was performed using logistic regression tests. Interactions among variables were studied with backward models based on likelihood ratios. Categorical variables were compared using chi-square test. Correlation tests (Spearman's correlation coefficient) were used to compare age and response to the drug. *p* values lower than 0.05 were considered statistically significant. All these analyses were carried out with SPSS 17.0. Both per-protocol and intention-to-treat analyses were performed.

## Results

### Study population and group assignments

**Figure 1:**
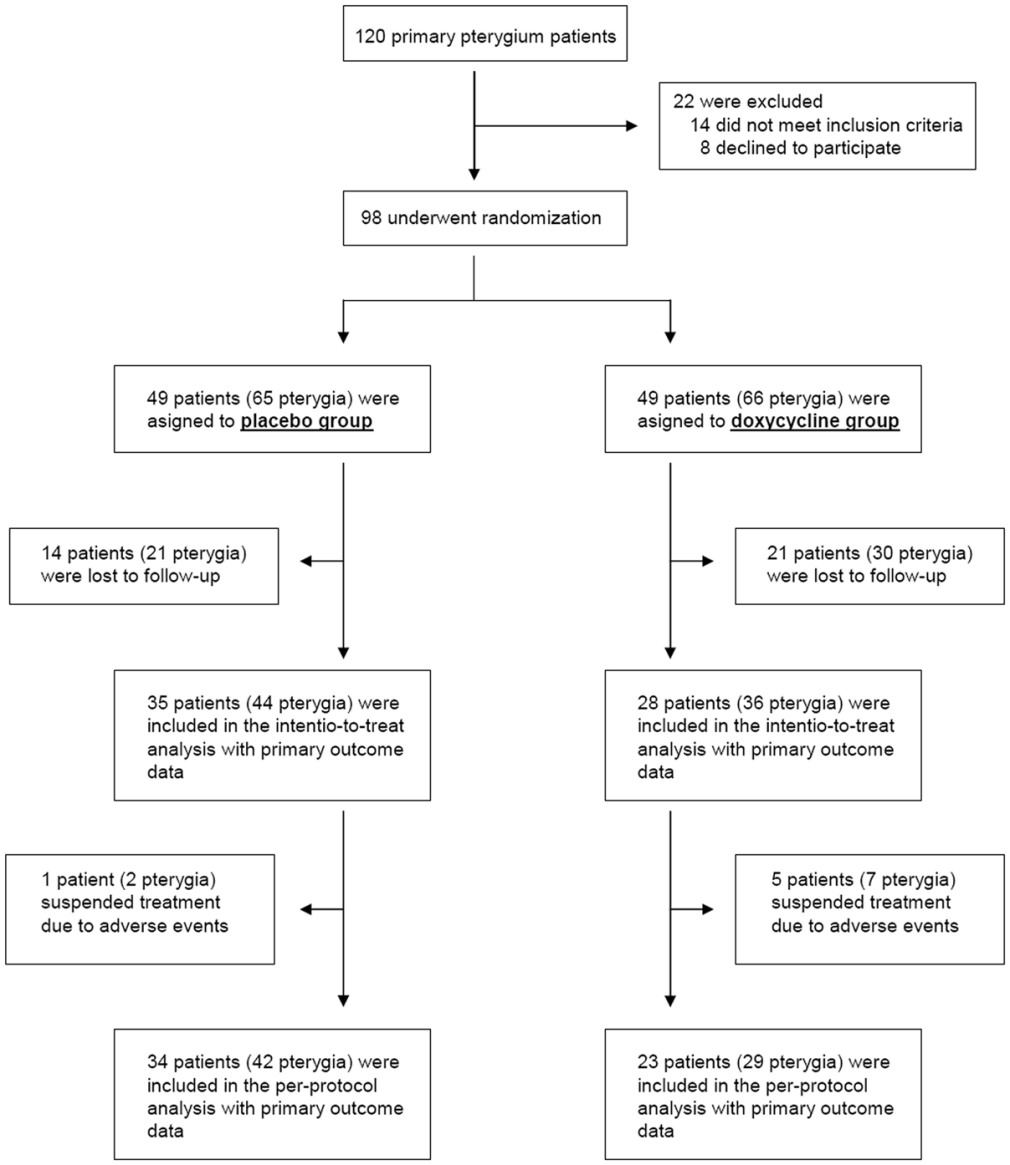
Consort flow diagram. The major reasons for not meeting the inclusion criteria were pregnancy (8) and being on a treatment with doxycycline incompatible drugs (6). Many patients were lost to follow-up probably due to the high number of immigrants, a very mobile population, that were enrolled in the trial. Six patients had to suspend treatment due to mild adverse events. Several patients had more than one pterygium, so the total number of pterygia is also included.

Between October 2009 and May 2010, a total of 98 patients diagnosed with primary pterygium underwent randomization (49 in each group) at the Hospital San Pedro (Logroño, Spain). In the per-protocol analysis, primary outcome data were available for 34 patients in the placebo group and 23 in the doxycycline-treated group (Fig. 1). Demographic and clinical characteristics at baseline were similar between the two groups (Table 1).

**Table 1:** Baseline demographic and clinical characteristics of the patients enrolled in the trial.

	Placebo (n = 49)	Doxycycline (n = 49)	Statistical test	*p* value
Sex (male/female)	28/21	27/22	? ^2^	1.00
Race or ethnic group* (Caucasian/Hispanic/Other)	19/24/6	19/25/5	? ^2^	0.95
Age† [range]	51.29±1.98 [29–80]	49.71±2.14 [20]–[86]	*t*	0.59
Initial size of pterygium‡ [range]	4.65±0.39 [1.07–14.48]	5.25±0.48 [0.71–17.49]	*t*	0.34
Initial pterygium morphology§ (T1/T2/T3)	(9/21/19)	(6/22/21)	? ^2^	0.69

* Race or ethnic group was self-reported.

† Plus-minus values are means ± E.

‡ Pterygium size is expressed in mm^2^.

§ As classified by Tan et al. [24].

### Adverse events

As expected for doxycycline, adverse events were minimal and resolved quickly after medication was suspended. These included 5 cases of nausea and vomit (1 placebo, 4 doxycycline), 2 cases of eczema (1 placebo, 1 doxycycline), and 1 case of phototoxicity (in the placebo group).

### Outcomes

**Figure 2:**
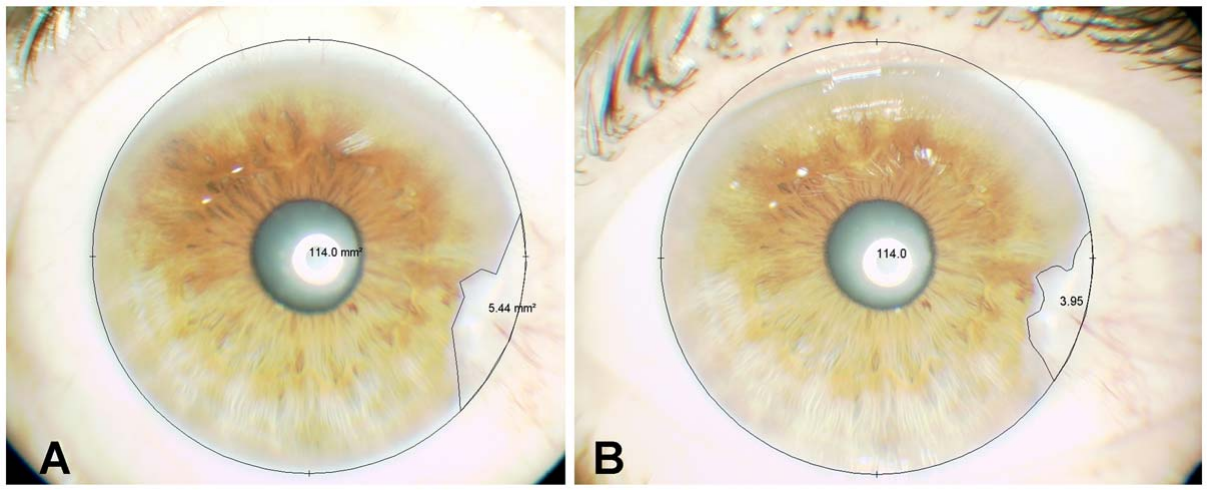
Photographs of the eye of patient number 20 (male, 61 year-old, Caucasian) before (A) and after (B) the treatment with doxycycline for 30 days. A clear reduction of the lesion's size can be appreciated, from 5.44 mm^2^ to 3.95 mm^2^.

Photographs of the pterygium lesions were taken in the first two visits (Fig. 2). After a month of treatment, the overall data showed that pterygia that had been treated with oral doxycycline experimented a relative change (size measured in the second visit divided by size in the first visit) of 0.98±0.05, whereas in those receiving placebo the relative change was 1.01±0.03. These differences were not statistically significant when analyzing either the per-protocol (*p* = 0.225) or the intention-to-treat (*p* = 0.191) populations (Student's *t* test).

Subgroup analysis was performed through logistic regression to investigate whether doxycycline had a beneficial effect on particular groups defined by age, sex, race, or initial lesion size and morphology. Pterygia whose relative change was ≥1 (did not change size or grew) were given the value “0” whereas those with a relative change <1 (growth reduction) were labeled as “1”. For race, patients of Caucasian origin were labeled “0”, and all others as “1”. Other variables included sex, age, initial pterygium morphology, and initial size of the pterygium lesion. A multivariant enter logistic regression (also called simultaneous regression) revealed than only “age” had an independent effect on lesion reduction (p<0.019). To test whether interactions occurred among all these variables, a backward logistic regression model, where all independent variables are entered at one time and then they are removed one at a time based on a preset significance value, based on likelihood ratios was performed (Table 2). On the third step, age, race, and treatment were the only significant variables. When we tried to estimate the particular odds ratios, the model did not converge due to insufficient sample size, thus only the statistical significance is shown. In summary, doxycycline effect on pterygium is heavily dependent on the race and age of the patient, being more efficient on people of Caucasian descent and older age.

**Table 2:** Statistically significant variables in the backward logistic regression model based on likelihood ratio.

Variable	Significance level
Treatment	<0.001
Age by treatment	<0.001
Race by treatment	0.002
Age by race by treatment	0.002

**Figure 3:**
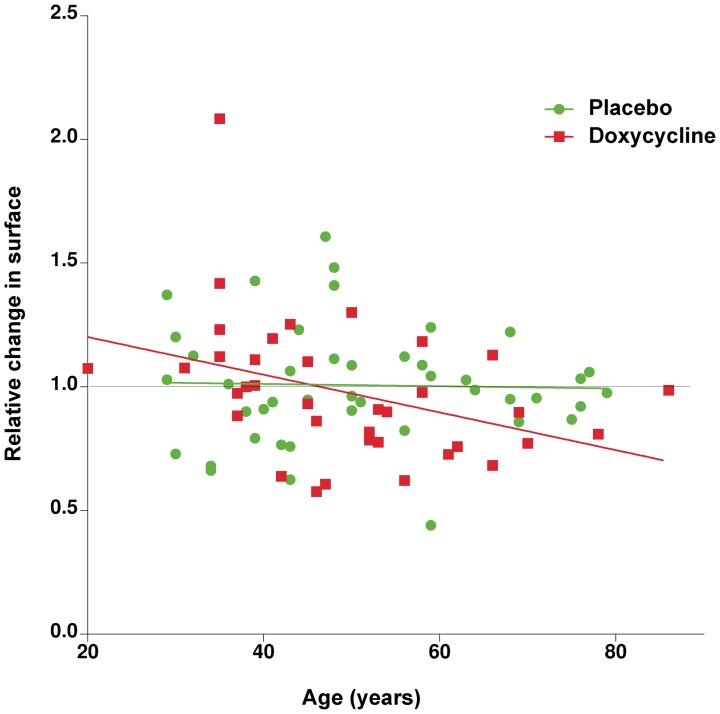
Correlation plot between patient's age (abscises) and relative response to treatment (ordinates) for patients that received either placebo (green circles) or doxycycline (red squares). The green and red lines represent the correlation slope for each set. There is a significant correlation for doxycycline treatment (Spearman's *r* = −0.4783, 95% CI −0.7025 to −0.1678, *p* = 0.003). Relative change in surface is the quotient between the size of the lesion in the second visit divided by the size in the first visit.

The effect of age on the efficacy of the doxycycline treatment was further investigated by correlation analysis. There was a positive correlation (Spearman's *r* = −0.4783, 95% CI −0.7025 to −0.1678, *p* = 0.003) between increasing age and larger reduction of the pterygium lesion on the treated individuals. Obviously, no such correlation occurred in patients receiving placebo (Fig. 3).

**Figure 4:**
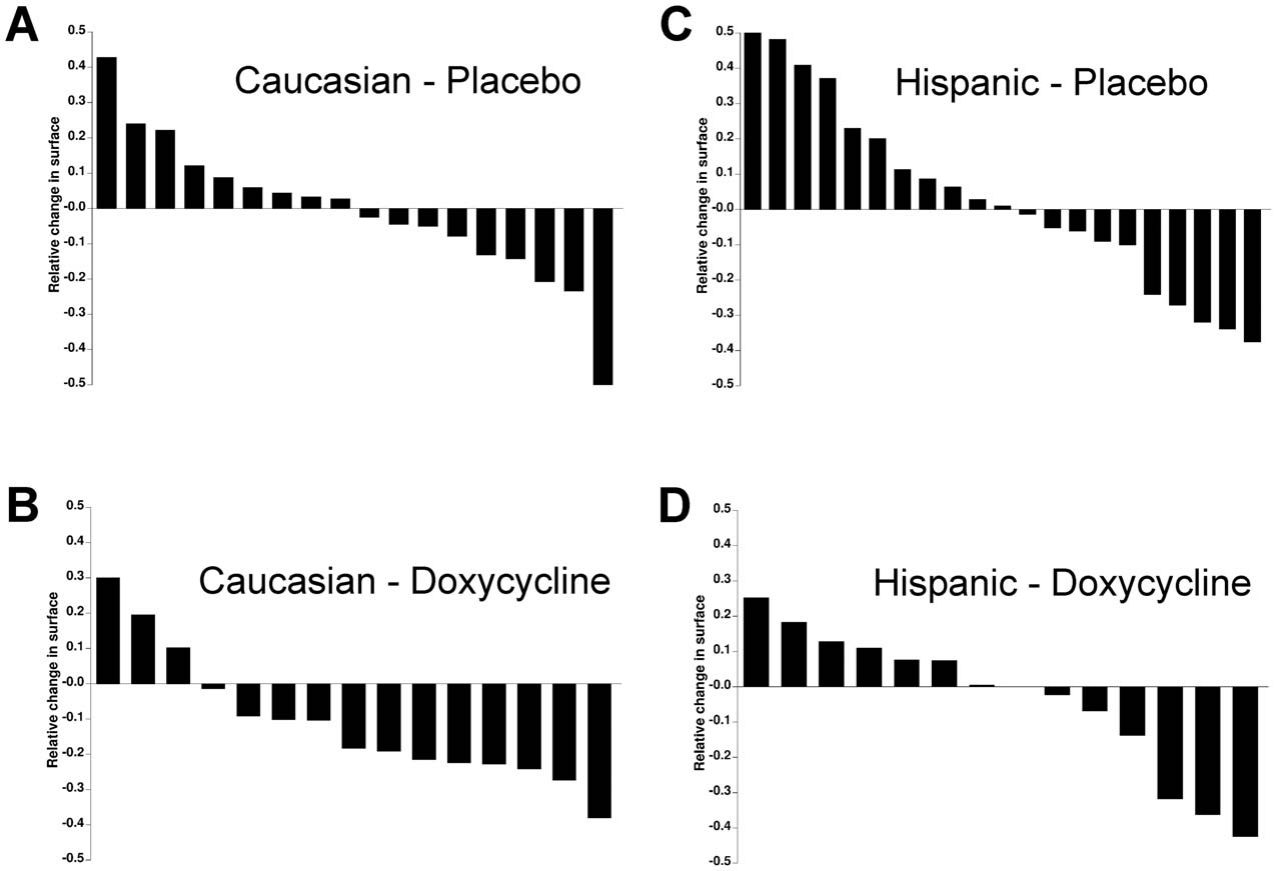
Waterfall representation of the relative changes in pterygium surface for all intention-to-treat patients treated with placebo (A, C) or with doxycycline (B, D), according to race. Each bar represents an individual pterygium. To comply with waterfall representation conventions, values are represented as relative change in surface minus 1, so that positive values represent lesion growth and negative values represent lesion reduction.

The contribution of race is represented through a waterfall graph (Fig. 4) where the effect of doxycycline is clearly seen in Caucasians (Fig. 4B) but is undistinguishable from placebo in Hispanic patients (Fig. 4D). Something unexpected was the wide variation in pterygium size we observed in the placebo-treated patients (Fig. 4 A, C). Only 15 patients underwent corrective surgery during the trial period and none of these presented a relapse during the 12 months following the end of the treatment, therefore not enough data was available to evaluate whether the treatment had any influence on recurrence frequency.

## Discussion

In this study we have shown that oral doxycycline, given during 30 days at 200 mg/day, does modify the growth pattern of pterygium lesions in Caucasian patients, a result which is in agreement with previous observations in a mouse model [21]. Moreover, older patients responded better to the treatment.

We were surprised by the low compliance seen in this trial. In fact, only 64% of patients came back for their second visit, which was needed to allow measurement of the primary outcome. Two reasons may explain this behavior. First, not being a life-threatening disease, pterygium is often viewed as a cosmetic problem rather than a serious condition. Second, pterygium is more prevalent in subtropical regions of the world, probably in relation with a higher UV light exposure [25]. In consequence, a large proportion of all diagnosed cases in Spain corresponds to South- and Central-American immigrants, which is a very mobile population, often depending on temporary employment. This issue should be taken into consideration for the design of future clinical trials on pterygium patients.

One of the most predictive variables of doxycycline response was race. Hispanic patients did not respond to doxycycline treatment whereas their Caucasian counterparts did. Broad differences have been reported in the prevalence of pterygium in various ethnic backgrounds [26]–[28]. There are also reports on different recurrence rates between Hispanic and white patients [29]. In addition, doxycycline has been shown to influence up to 332 genes when applied to pterygium cells, involving relevant cellular pathways such as mitochondrial gene expression, endoplasmic reticulum stress, integrins, extracellular matrix components, cell cycle regulators, and growth factors [21]. Any differences in theses pathways among races may be responsible for their differential responses to the drug. Gene profiling studies comparing pterygia originated in Caucasian and Hispanic patients may shed some light on this unexpected behavior. In addition, studies can be designed for the study of pterygium tissue collected after surgical removal to see the differences elicited by doxycycline treatment in these patients.

There is a significant correlation between pterygium incidence and age [28], [30], [31] but an explanation is needed for the observation that doxycycline treatment was more efficient in older patients. Perhaps the eye of older patients becomes more permeable to systemic doxycycline, thus allowing a higher dosage on the eye surface. This may be in connection with the fact that the beneficial effect of doxycycline was much larger in mice [21] than in humans, which may be due to several reasons. First of all, the anatomy and physiology of mouse and human eyes are very different [32]. These differences include size, diurnal vs. nocturnal habits, and histological features such as a rudimentary Bowman's membrane in the mouse [33], and might influence the pharmacokinetics of an orally administered drug. There are some discrepancies on the amount of orally administered doxycycline that can reach the eye surface. For instance, in a set of patients and healthy individuals which were given oral doxycycline, the drug was easily found in the blood but the tear film was devoid of it [34]. A similar result was found in horses [35]. Nevertheless, an experiment performed in macaques found that oral administration of doxycycline was enough to induce transgene activation in the eye of these animals [36]. In the same direction, oral doxycycline was able to reduce angiogenesis in the rat cornea [19]. Therefore, there seems to be an inverse correlation between eye size and doxycycline ability to reach the outer surface of the eye. Other antibiotics encounter also some difficulties to cross the hemato-ocular barrier in humans [37]. There may be an age-dependent relaxation of this barrier or just an increase on doxycycline sensitivity in older patients.

Another unexpected observation was the ample variation in pterygium size modifications seen in placebo-treated patients. A small part of this variation may be due to measurement errors and the difficulty to properly measure the size of pterygium lesions has been previously recognized [38]. But, apart from that, there were real changes in size in the four weeks between photographs, including both increases and decreases of the total affected area. This may represent an intrinsic feature of the biology of pterygium which had not been previously recognized. There may exists an unstable equilibrium between factors promoting pterygium growth and host defence mechanisms trying to reduce it, which results in this “wobbling” behavior. This fact should be taken into consideration for the design of future clinical trials.

In conclusion, oral doxycycline reduces pterygium lesion size in white patients and could be used as a treatment for pterygium symptoms or as a means to delay surgical resection. In this trial we have used a systemic treatment to follow our previous experience on an animal model [21] but topical application of doxycycline may be even more efficient in controlling pterygium size. Larger clinical trials are needed to better characterize this effect and to investigate the efficiency of doxycycline on other ethnic backgrounds.

## Supporting Information

Checklist S1 **CONSORT Checklist.**
(DOC)

Protocol S1 **Trial protocol** (**in Spanish**)**.**
(DOC)

Protocol S2 **Trial protocol** (**in English**)**.**
(DOC)
