# Supplementary material for: Oral Doxycycline Reduces Pterygium Lesions; Results from a Double Blind, Randomized, Placebo Controlled Clinical Trial
Source: PLoS One. 2012 Dec 19;7(12):e52696. doi: 10.1371/journal.pone.0052696 (PMC3526544; doi:10.1371/journal.pone.0052696)
Supplement: Protocol S1 — Trial protocol (in Spanish). (DOC) [file pone.0052696.s002.doc]

| **Estudio unicéntrico de fase II, doble ciego, randomizado, comparando doxiciclina administrada oralmente frente a placebo en pacientes con pterigión** |
| --- |

**PROMOTOR** Centro de Investigación Biomédica de La Rioja (CIBIR)

C/ Piqueras 98

26006 Logroño (La Rioja)

Tel: 941 278 775

**PRODUCTO** Doxiciclina (Vibracina ®, 100 mg)

**PROTOCOLO** Versión 3 (Fecha: Abril 2009)

| COMPROMISO DE CONFIDENCIALIDAD |
| --- |
| Este documento no puede ser reproducido, transmitido o copiado de ninguna forma, en su totalidad o parcialmente, ni su contenido revelado a terceras partes sin la previa aprobación escrita del promotor. |

**CONTENIDO**

A.- Introducción…………….……………………………………………….………….. 3

B.- Diseño ……………………………………………………………………………… 5

C.- Objetivos …………………………………………………………………………… 7

D.- Fármaco experimental ……………………………………………………………... 8

E.- Pacientes ……………………………………………………………………….…… 9

F.- Análisis estadístico ……………………………………………………….………... 12

G.- Calendario ………………………………………….…………………….…….….. 13

H.- Seguridad y consideraciones éticas ……………………………………….……….. 14

**A.- Introducción e hipótesis**

El pterigión es un tumor benigno que crece en la conjuntiva y en la córnea con problemas obvios para la visión del paciente. Histológicamente, la lesión se caracteriza por metaplasia del epitelio plano, hiperplasia de células caliciformes y expresión anormal de p53. Este epitelio se apoya en un tejido conjuntivo rico en fibroblastos y vasos sanguíneos. Estas células transformadas expresan gran cantidad de factores que aumentan su capacidad angiogénica y migratoria, incluyendo entre otros VEGF, versican, TGFβ, IL-6, IL-8, etc. (Di Girolamo et al. Pathogenesis of pterygia: role of cytokines, growth factors, and matrix metalloproteinases. Prog. Retin. Eye Res. 2004; 23:195-228).

La intervención terapéutica habitual implica la extirpación quirúrgica de la lesión, pero el porcentaje de recidivas es muy alto. La segunda intervención quirúrgica causa habitualmente serios problemas en la visión del paciente debido a la presencia de cicatrices y otros problemas secundarios (Dushku et al. Pterygia pathogenesis: corneal invasion by matrix metalloproteinase expressing altered limbal epithelial basal cells. Arch. Ophthalmol. 2001; 119:695-706).

La doxiciclina es un antibiótico bacteriostático que ha sido utilizado en clínica durante décadas. Se ha demostrado su capacidad de inhibir la angiogénesis, a través de una inhibición de las metaloproteasas, mediante un mecanismo independiente de su capacidad antimicrobiana (Dan et al. Inhibitory effect of oral doxycycline on neovascularization in a rat corneal alkali burn model of angiogenesis. Curr. Eye Res. 2008;33:653-660).

En un experimento reciente, utilizando un modelo de pterigión *in vivo*, se ha demostrado que la doxiciclina por sí sola es capaz de eliminar el pterigión en ratones con una eficacia del 100% (Cox et al. Doxycycline inhibits pterygium cell lesions and ocular angiogenesis. Invest. Ophthalmol. Vis. Sci. , en prensa).

Evidentemente, la hipótesis que se intenta demostrar en este estudio es que un tratamiento con doxiciclina es capaz de reducir el crecimiento del pterigión en pacientes. En el caso de que no sólo reduzca sino que elimine esta lesión, como sucede en ratones, los beneficios para los pacientes como para el Hospital son obvios, ya que se evitaría una intervención quirúrgica con todo lo que ello implica de riesgos para el paciente, gastos de quirófano y horas de utilización del mismo.

Por lo tanto se trata de una hipótesis de superioridad del tratamiento con doxiciclina frente a la no intervención, previa al tratamiento quirúrgico.

Esta será la primera vez que se utilice la doxiciclina en humanos para tratar el pterigión.

**B.- Diseño del Ensayo Clínico**

Se ha diseñado el estudio como un ensayo unicéntrico, a desarrollar en el Hospital San Pedro de Logroño (La Rioja). Se estudiarán dos grupos, uno en que se suministrará doxiciclina (2 dosis diarias de 100 mg cada una) durante 30 días y otro en que se suministrará placebo. Los pacientes se asignarán a uno de los dos brazos aleatoriamente siguiendo una proporción 1:1 (tratamiento:placebo). El enmascaramiento se hará mediante una técnica de doble ciego, en que ni el paciente ni el médico sabrán en ningún momento quien recibe tratamiento y quien placebo.

Para completar el ensayo, cada paciente deberá acudir a 4 visitas según el siguiente plan:

Visita 1. Se tomarán datos para la historia clínica y se hará el diagnóstico de la lesión. Si el paciente cumple los criterios de inclusión y no presenta ninguno de los criterios de exclusión se le pedirá consentimiento informado para participar en el ensayo. Si el paciente consiente, se le asignará un código por el que será identificado durante todo el ensayo. Se tomará una fotografía del ojo dañado (o de ambos, si ambos tuvieran la lesión) que se identificará con el código del paciente. Se le entregarán las cápsulas, preparadas por el Servicio de Farmacia del Hospital San Pedro, marcadas con su código (que habrán sido previamente distribuidas al azar) y se le darán instrucciones de cómo tomarlas y cuándo debe volver para las siguientes visitas (hoja de información al paciente).

Con los pacientes excluidos o que no den su consentimiento para participar en el ensayo se seguirán los protocolos habituales establecidos en el Servicio de Oftalmología para el tratamiento de su enfermedad.

Visita 2. Se hará 30 días después de la Visita 1. Se evaluará el grado de cumplimiento del tratamiento anotando cuántas cápsulas no se han tomado a lo largo de los 30 días de tratamiento. Se tomará una segunda fotografía del ojo enfermo para poder comparar con la fotografía de la primera visita. La fotografía se marcará de nuevo con el código del paciente. El médico volverá a reconocer al paciente y, dependiendo del estadio de la lesión, tomará la decisión de intervenir o no quirúrgicamente al paciente siguiendo los protocolos habituales del Servicio de Oftalmología.

Visita 3. Tendrá lugar 6 meses después de la Visita 2. El médico reconocerá al paciente y anotará sus observaciones. En especial se tendrá en cuenta si ha habido una recidiva del pterigión.

Visita 4. Tendrá lugar 6 meses después de la Visita 3. El médico reconocerá al paciente y anotará sus observaciones. En especial se tendrá en cuenta si ha habido una recidiva del pterigión.

**C.-** **Objetivos y variables de valoración**

El objetivo principal es reducir el crecimiento del pterigión mediante un tratamiento farmacológico y reducir el número de casos que deben ser extirpados quirúrgicamente.

Como objetivos secundarios tenemos:

1.- Determinar si el tratamiento con doxiciclina reduce la probabilidad de recidiva en pacientes operados de pterigión.

2.- Investigar qué genes y/o proteínas cambian su expresión después del tratamiento del pterigión con doxiciclina.

Para cumplir estos objetivos, se tomará una fotografía del pterigión en la primera visita. Después de 30 días de tratamiento se tomará una segunda fotografía de la misma zona. Se medirá la superficie ocupada por el pterigión en ambas fotografías utilizando un software de procesado de imágenes. La variable principal de valoración será el cociente entre la superficie ocupada en la segunda fotografía y la de la primera fotografía (tasa de crecimiento o reducción, según cada caso).

Como variables secundarias se analizará la tasa de recidiva a 12 meses después de la intervención quirúrgica y la expresión génica/proteómica del pterigión extirpado.

**D.-** **Fármaco experimental**

La doxiciclina es un antibiótico tetraciclínico de larga duración derivado de la oxiciclina, siendo posible su administración en una sola dosis al día. La doxiciclina se puede administrar por vía intravenosa, oral, subgingival e intragingival. Después de una administración oral, la doxiciclina se absorbe en un 90-100%. La absorción es retrasada cuando el fármaco se administra con alimentos o con leche. De la misma forma, los antiácidos a base de sales de aluminio y las sales de hierro reducen la absorción de forma significativa. La doxiciclina atraviesa la barrera placentaria y aparece en la leche materna. La mayor parte del fármaco se excreta en las heces, siendo mínimamente eliminada por vía renal. En el ensayo se suministrará en cápsulas de 100 mg preparadas por la Farmacia del Hospital. Los pacientes tomarán dos cápsulas diarias ya que se ha comprobado que la vida media de este compuesto en sangre es de 16 horas (Archer et al. Treatment and prophylaxis of bacterial infections. In: Fauci et al. Editors. Harrison´s principles of internal medicine. 14th ed. New Cork:McGraw-Hill; 1998, pp 856-869). Por otra parte, se ha demostrado que ésta es la dosis óptima para conseguir una inhibición significativa de los enzimas que digieren la matriz extracelular (Smith et al. Oral administration of doxycycline reduces collagenase and gelatinase activities in extracts of human osteoarthritic cartilage. J. Rheumatol. 25:532-535; 1998). Además, el uso de esta dosis durante largos periodos de tiempo, de hasta 30 meses, produce muy pocas reacciones adversas en los pacientes (Brandt et al., Effects of doxycycline on progression of osteoarthritis. Results of a randomized, placebo-controlled, double blind trial. Arthritis Rheum. 52:2015-2025; 2005).

El brazo control recibirá cápsulas idénticas rellenas de excipiente como placebo.

El placebo será fabricado por el Servicio de Farmacia del Hospital San Pedro y las cápsulas de Vibracina ® (doxiciclina) serán extraídas de los blisters y reenvasadas en frascos topacio, conteniendo cada uno las 60 cápsulas prescritas para todo el tratamiento. Se adjuntan los PNT de fabricación y reenvasado.

**E.-** **Pacientes**

**Número total de pacientes**

Hay dos formas de calcular el tamaño de la muestra necesaria para alcanzar significación estadística en el ensayo. La primera es una estrategia cualitativa donde cada paciente es clasificado como éxito o fracaso ante el tratamiento dependiendo de que su pterigión disminuya por debajo de un valor determinado a priori (valor de corte) o no. Esto permite la comparación de dos proporciones mediante una prueba χ^2^ bilateral para dos muestras independientes. Del estudio de la patofisiología de la enfermedad sabemos que el pterigión no tratado tiende a crecer o a mantenerse con el mismo tamaño, pero nunca a reducirse espontáneamente por lo que podemos asumir que la proporción en el grupo tratado con placebo es asintóticamente 0,0%. Dependiendo de la potencia y de los valores de corte establecidos, el número de pacientes que habría que reclutar en cada grupo son los siguientes:

| **Potencia** | **Corte en 25%** | **Corte en 50%** | **Corte en 75%** | **Corte en 100%** |
| --- | --- | --- | --- | --- |
| 80% | 27 | 11 | 6 | 1 |
| 90% | 35 | 14 | 7 | 2 |

*Los números indican el tamaño muestral en cada grupo

En segundo lugar, también podemos utilizar una estrategia cuantitativa en que cada paciente recibe un valor numérico que representa el porcentaje de reducción de su pterigión debido al tratamiento. En este caso, para detectar diferencias significativas en el contraste de la hipótesis nula Ho:1=2 se utiliza una prueba t-Student bilateral para dos muestras independientes. Si asumimos que el promedio de reducción de la extensión del pterigión en el grupo placebo es de 5 puntos porcentuales y en el grupo tratado con doxiciclina es de 15 puntos porcentuales y que la desviación típica de ambos grupos es como máximo de 15 puntos porcentuales, y para un nivel de significación de 5%, el número de sujetos dependiendo de la potencia es el siguiente:

| **Potencia** | **Número de pacientes por grupo** |
| --- | --- |
| 80% | 35 |
| 90% | 49 |

Ya que es difícil prever el grado de respuesta esperado, nos hemos decidido por el escenario que puede arrojar más información y se reclutarán 98 pacientes (49 tratados con doxiciclina y 49 tratados con placebo). Este tamaño muestral permitirá detectar como significativas diferencias de 10 puntos porcentuales o más en la disminución del tamaño del pterigión entre el grupo tratado y el grupo control. Además este número de pacientes también permitirá contrastar los diferentes puntos de corte de 25%, 50%, 75% y 100%.

El reclutamiento se efectuará entre los pacientes que acudan al Servicio de Oftalmología del Hospital. Actualmente acuden al Servicio entre 4 y 8 pacientes por mes aquejados de esta enfermedad.

**Criterios de inclusión**

1. Antes del comienzo de los procedimientos especificados en el protocolo, deberá obtenerse y documentarse el consentimiento informado.
2. Pacientes con pterigión primario no intervenido que produzca alguno de los siguientes síntomas:
   1. astigmatismo sin otra causa.
   2. Sensación de cuerpo extraño.
   3. Afectación corneal amenazando el eje visual.
3. Edad ≥ 18 años.
4. Capaz de cumplir con el seguimiento programado.

**Criterios de exclusión**

1. Mujeres embarazadas, en periodo de lactancia, o que estando en edad fértil no sigan un plan anticonceptivo.
2. Alergia a doxiciclina o presencia de enfermedades que contraindiquen el empleo de doxiciclina como lupus eritematoso sistémico o miastemia gravis.
3. Toma de medicamentos incompatibles con doxiciclina o que mengüen la eficacia/absorción de ésta.
4. Pacientes que no puedan ser seguidos regularmente por razones psicológicas, sociales, familiares o geográficas.

**F.-** **Análisis estadístico**

Las variables que se someterán a análisis estadístico son, por una parte, el crecimiento o reducción de la superficie ocupada por el pterigión en relación con la superficie observada al comienzo del tratamiento. Además se analizará el porcentaje de recidiva en ambos brazos y la expresión de distintos genes y proteínas comparando los pterigión tratados y los no tratados.

En primer lugar, se distribuirá a los pacientes dependiendo del grado de reducción de su pterigión en el grupo de éxito (aquellos cuya reducción sea mayor de un valor determinado) o fracaso (aquellos que no alcancen dicha reducción) y se analizará la hipótesis nula mediante un test de χ^2^ bilateral para dos muestras independientes (análisis cualitativo). Se utilizarán distintos niveles de corte. También se hará un análisis cuantitativo de los datos. En principio, se comprobará la distribución de los datos y su varianza. Si estos datos son homocedásticos y presentan una distribución normal, la comparación entre los dos grupos se puede realizar mediante herramientas simples tales como el test de la *t* de Student. De lo contrario se utilizarán tests no paramétricos. Se considerará que las diferencias son estadísticamente significativas cuando el valor de *p* sea inferior a 0,05. Se incluirán en el análisis todos los pacientes que acudan a la segunda entrevista y hayan seguido el tratamiento prescrito y, por lo tanto, que dispongamos de fotografías de su pterigión antes y después del tratamiento (análisis por tratamiento finalizado). También se realizará un análisis de todos los pacientes reclutados, independientemente de si han finalizado o no el tratamiento (análisis por intención de tratar).

**G.-** **Calendario previsto**

Cada paciente concreto permanecerá formalmente en el ensayo clínico durante 13 meses. Tendrá una primera visita donde se tomarán todos los datos pertinentes y se le informará de la dinámica del ensayo. En esta primera visita se tomará una fotografía del globo ocular afectado por el pterigión y se le proporcionarán los 60 comprimidos que debe tomar durante los 30 días de tratamiento. Al cabo de los 30 días se tendrá otra entrevista en la que se recogerán datos del grado de cumplimiento del tratamiento y se tomará una segunda fotografía del globo ocular. En este momento se determinará si el paciente requiere cirugía para extirpar el pterigión y se procederá a llevarla a cabo según los protocolos del Servicio de Oftalmología, realizándose con la misma técnica en todos los pacientes y realizándose por un máximo de dos cirujanos. Habrá dos visitas más, a los 6 y 12 meses después de la segunda visita donde básicamente se evaluará si hay signos de recidiva del pterigión, en cuyo caso se aplicarán las terapias adecuadas según los protocolos del Servicio de Oftalmología.

En cuanto al ensayo en sí, calculamos un periodo de reclutamiento de aproximadamente 18 meses para conseguir los 98 pacientes necesarios. Si a esto se añaden los 13 meses para que el último de los pacientes cumpla su seguimiento, el cierre del ensayo tendría lugar a los 31 meses de su comienzo. La fecha prevista de comienzo es el 1 de febrero de 2009 y la fecha de cierre es el 31 de agosto de 2011.

**H.-** **Seguridad y consideraciones éticas**

La doxiciclina es un fármaco con un perfil de seguridad muy alto y bien conocido.

Un tratamiento con doxiciclina a corto plazo presenta riesgos muy bajos ya que se trata de un medicamento que se ha utilizado en la clínica durante décadas sin que se hayan comunicado efectos adversos que desaconsejen su uso (Brandt et al., Effects of doxycycline on progression of osteoarthritis. Results of a randomized, placebo-controlled, double blind trial. Arthritis Rheum. 52:2015-2025; 2005). Si el tratamiento funciona de forma similar a lo mostrado en los estudios preclínicos en ratones, los pacientes podrán beneficiarse de una reducción notable de su pterigión, pudiendo llegar incluso a no necesitar intervención quirúrgica.

En los sujetos que reciban placebo no se prevé ningún beneficio concreto pero tampoco saldrán perjudicados ya que lo único que se les pide es retrasar 4 semanas la posible intervención quirúrgica que extirpe su pterigión. En este periodo de tiempo el crecimiento de la lesión es mínimo.

No se prevé involucrar a poblaciones vulnerables.

No están previstas compensaciones económicas.

La información que se dará a los sujetos participantes, sus familiares y representantes legales está recogida en los Anexos (Hoja de Información a los pacientes).

No habrá exploraciones adicionales. Se dará el cuidado habitual a los pacientes aquejados de pterigión.

Se facilitará, en el caso de que así se considere por la Agencia o Institución pertinente, las monitorizaciones, auditorias, revisiones del CEIC e inspecciones reguladoras relacionadas con el ensayo, facilitando el acceso directo a los documentos / datos originales.

Se utilizará placebo como control, ya que no existe ningún tratamiento farmacológico aprobado para el tratamiento del pterigión.
