# Supplementary material for: Oral Doxycycline Reduces Pterygium Lesions; Results from a Double Blind, Randomized, Placebo Controlled Clinical Trial
Source: PLoS One. 2012 Dec 19;7(12):e52696. doi: 10.1371/journal.pone.0052696 (PMC3526544; doi:10.1371/journal.pone.0052696)
Supplement: Protocol S2 — Trial protocol (in English). (DOC) [file pone.0052696.s003.doc]

| **Randomized, double blind, unicentric, phase II clinical trial, comparing oral doxycycline versus placebo in pterygium patients** |
| --- |

**PROMOTOR** Centro de Investigación Biomédica de La Rioja (CIBIR)

C/ Piqueras 98

26006 Logroño (La Rioja)

Tel: 941 278 775

**PRODUCT** Doxycycline (Vibracina ®, 100 mg)

**PROTOCOL**  Version 3 (Date: April 2009)

**CONTENTS**

A.- Introduction…………….……………………………………………….………….. 3

B.- Design ……………………………………………………………………………… 5

C.- Objectives ……………………………………………………..…………………… 7

D.- Experimental drug ……..…………………………………………………………... 8

E.- Patients …………..………………………………………………………………… 9

F.- Statistical analysis ………..……………………………………………………….. 12

G.- Time table ………………………………………….…………………………….. 13

H.- Security and ethical considerations …………………………...………………….. 14

**A.- Introduction and hypothesis**

Pterygium is a benign tumor growing in the conjunctiva and the cornea resulting in obvious problems for patients´ sight. Histologically, the lesion is characterized by metaplasia of the squamous epithelium, goblet cell hyperplasia and abnormal expression of p53. Under the epithelium, the connective tissue contains numerous fibroblasts and blood vessels. The malignant cells express large amounts of factors which result in increased angiogenesis and migration capabilities, these include VEGF, versican, TGFβ, IL-6, and IL-8, among others (Di Girolamo et al. Pathogenesis of pterygia: role of cytokines, growth factors, and matrix metalloproteinases. Prog. Retin. Eye Res. 2004; 23:195-228).

The usual therapy includes the surgical resection of the lesion, but the percentage of relapses is very high. If a second intervention is needed, this may cause serious vision problems due to the formation of scar tissue and other side effects (Dushku et al. Pterygia pathogenesis: corneal invasion by matrix metalloproteinase expressing altered limbal epithelial basal cells. Arch. Ophthalmol. 2001; 119:695-706).

Doxycycline is a bacteriostatic antibiotic that has been used in the clinic for decades. It has been shown that doxycycline is able to block angiogenesis through a metalloproteinase inhibition mechanism which is independent of its antimicrobial activity (Dan et al. Inhibitory effect of oral doxycycline on neovascularization in a rat corneal alkali burn model of angiogenesis. Curr. Eye Res. 2008;33:653-660).

In a recent experiment, using an animal model of pterygium in vivo, it was shown that doxycycline by itself was able to eliminate pterygium in mice with a 100% efficacy (Cox et al. Doxycycline´s effect on ocular angiogenesis: an in vivo analysis. Ophthalmology, in press).

The hypothesis we are trying to test in this study is that a doxycycline treatment may be able to reduce pterygium growth in patients. In case the lesions are totally eliminated, as happens in mice, the benefits would be obvious for both the patients and the Health system, since surgical interventions would be reduced with all the implications in patients´ safety, and economic savings in operating room occupation and running costs. If the reduction were partial, then doxycycline could be used to delay surgery when needed.

Therefore, this a hypothesis of superiority of the doxycycline treatment versus a placebo in the growth of pterygium.

This is the first time doxycycline is used in humans to treat pterygium.

**B.- Clinical Trial Design**

The study has been designed as a unicentric trial, to be carried out at the Hospital San Pedro, Logroño (La Rioja), Spain. Two groups will be studied, one will receive doxycycline (2 daily doses, 100 mg each) for 30 days, while the other will receive a placebo. Patients will be randomly assigned to one of the two arms following a 1:1 proportion (treatment:placebo). Masking will be done using a double blind technique, so neither the patient nor the doctor will know who is receiving doxycycline or placebo.

To complete the study, each patient will be asked to attend 4 visits to the clinic, as follow:

Visit 1. Clinical history data will be collected and the lesion will be properly diagnosed. If the patient meets the inclusion criteria and does not fulfil any of the exclusion criteria, he/she will be asked to participate in the trial and sign the informed consent form. Consenting patients will be given a code for identification throughout the study. A photograph of the affected eye (or both in bilateral lesions) will be taken. The Pharmacy of the Hospital will provide the patient with a capsule bottle (randomly assigned to his/her study code containing either doxycycline or placebo) and the proper instructions to take the medicine together with instructions for following visits and telephone numbers for potential adverse affects.

If a patient needs to be excluded or does not consent to be included in the study, he/she will be asked for informed consent to use cells from his/her resected pterygium for experiments on cell and molecular biology. All regular procedures will be followed with these patients.

Visit 2. This will happen 31 days alter the first visit. The degree of compliance will be evaluated and the number of capsules that were not taken for any reason will be recorded. A second photograph of the affected eye(s) will be taken to compare with that taken in the first visit. The ophthalmologist will again evaluate the lesion and, depending on the stage of this lesion, a decision will be taken to remove it or not, following the usual protocols set in place by the Ophthalmology Service. If the pterygium is removed, the tissue will be sent to the CIBIR for cellular and molecular analyses.

Visit 3. It will take place 6 months alter the second one. The doctor will recognize the patient and record his/her observations. Particular care will be devoted to annotate any recurrences.

Visit 4. It will take place 6 months alter the third one. The doctor will recognize the patient and record his/her observations. Particular care will be devoted to annotate any recurrences.

**C.-** **Objectives and outcome endpoints**

The main objective of the study is to reduce pterygium growth with a pharmacological treatment and reduce the Lumber of cases that need surgical resection of the lesion.

As secondary objectives we look forward to:

1.- Determine whether treatment with doxycycline reduce recurrence rates in patients whose pterygium was surgically removed.

2.- Investigate which genes and/or proteins change their expression following treatment of pterygium cells with doxycycline.

To accomplish these objectives, a photograph of the lesion(s) will be taken Turing the first visit. After 30 days of treatment, a second photography of the same area will be taken. The surface occupied by the lesion will be calculated in both photographs using an image analyzing software package. The main outcome will be the ratio between the surface occupied by the lesion in the second photograph divided by the size in the first one (growth rate).

As secondary outcomes, we will analyze the recurrence rate at 12 months after the intervention and the genomic/proteomic expression of the resected pterygium.

**D.-** **Experimental drug**

Doxycycline is a long lasting tetracyclinic antibiotic derived from oxycycline, whose administration can be performed in a single daily dose. Doxycycline can be administered in different ways: orally, intravenously, subgingivally, and intragingivally. Following oral administration, 90-100 % doxycycline gets absorbed. Absorption is delayed when this drug is administered together with food or milk. In the same way, antiacids containing aluminium or iron salts significantly reduce absorption. Doxycycline crosses the placental barrier and is present in mothers´ milk. Most of the drug is excreted in the faeces, with very little being eliminated through the kidney. In the study doxycycline will be provided as 100 mg capsules, prepared by the Hospital pharmacy. Patients will take 2 capsules a day since the half life of this drug has been calculated at 16 hours (Archer et al. Treatment and prophylaxis of bacterial infections. In: Fauci et al. Editors. Harrison´s principles of internal medicine. 14th ed. New Cork:McGraw-Hill; 1998, pp 856-869). In addition, this dosis is optimal for significantly inhibiting the enzymes that degrade the extracellular matrix (Smith et al. Oral administration of doxycycline reduces collagenase and gelatinase activities in extracts of human osteoarthritic cartilage. J. Rheumatol. 25:532-535; 1998). Moreover, the use of this dosage for long periods of time, up to 30 months, produces very few and moderate adverse effects in patients (Brandt et al., Effects of doxycycline on progression of osteoarthritis. Results of a randomized, placebo-controlled, double blind trial. Arthritis Rheum. 52:2015-2025; 2005).

The control Group will receive identical capsules filled up with placebo (sugar pill).

Those patients excluded from the formal trial will be asked for permission (through informed consent) to use their excised pterygium tissue to establish cell cultures and perform additional cell and molecular biology studies.

**E.-** **Patients**

**Total Lumber of patients**

There are 2 ways of calculating sample size to reach statistical significance in a clinical trial. The first one is a qualitative strategy where each patient is classified as a success or a failure to the treatment depending on whether their lesion size shrinks below a predetermined value (threshold). This allows a comparison between groups using a bilateral χ^2^ for 2 independent samples. From the study of the disease we know that untreated pterygia keeps growing or maintains its size, but never tends to spontaneously reduce its size, so we can assume that the proportion of the placebo-treated group is asymptotically 0.0%. Depending of the power and the established threshold, the number of patients for each group are the following:

| **Power** | **Treshold at 25%** | **Treshold at 50%** | **Treshold at 75%** | **Treshold at 100%** |
| --- | --- | --- | --- | --- |
| 80% | 27* | 11 | 6 | 1 |
| 90% | 35 | 14 | 7 | 2 |

* Numbers indicate sample size on each group.

Secondly, we can also use a quantitative strategy where each patient receives a numeric value representing the percentage reduction (or growth) of his/her pterygium due to treatment. In this case, to detect significant differences in the null hypothesis contrast Ho:μ1=μ2 we will use a bilateral Student´s *t* test for 2 independent samples. If we assume that the mean reduction of the lesion in the placebo group is 5 % and in the doxycycline-treated group is 15 %, and that the standard deviation in both groups is a maximum of 15%, for a significance levels of 5%, the number of subjects is as follows:

| **Power** | **Number of patients per group** |
| --- | --- |
| 80% | 35 |
| 90% | 49 |

Since it is difficult to forecast the expected response, we have decided to follow the most stringent conditions and 98 patients will be recruited (49 treated with doxycycline and 49 treated with placebo). This sample size will allow to detect significant differences of 10% or higher in the diminution of pterygium size between the treated and the placebo groups. Besides, this number of patients will allow the qualitative analysis previously explained.

Recruitment will be performed among patients attending the Ophthalmology Service of the Hospital. Currently, 4 to 8 pterygium patients visit the Hospital every month.

**Inclusion criteria**

1. Before starting any of the procedures specified in this protocol, informed consent forms must be signed by the patients.
2. Patients with untreated primary pterygium producing at least one of the following symptoms:
   1. Astigmatismo with no other cause.
   2. Foreign body sensation.
   3. Corneal affectation theatening the visual axis.
3. Age ≥ 18 years.

4. Able to comply with the treatment and the follow-up visits.

**Exclusion criteria**

1. Pregnant or lactating women, women in fertile age not following an anticonceptive plan.
2. Alergies to doxycycline or presence of other diseases where doxycycline might be contraindicated, such as lupus eritematosus or miastemia gravis.
3. Patients taking drugs that are incompatible with doxycycline or that reduce doxycycline efficacy.
4. Patients that can not be followed up regularly by psychological, social, familial, or geographical reasons.

Those patients excluded from the formal trial will be asked for permission (through informed consent) to use their excised pterygium tissue to establish cell cultures and perform additional cell and molecular biology studies.

**F.-** **Statistical analysis**

The variables that will be statistically analyzed are, on the one hand, the growth or reduction of the surface occupied by the pterygium lesion after the treatment as compared with that before the treatment. In addition, the recurrence percentage in both arms and the expression of genes and proteins in pterygium cells either treated or not with doxycycline will be analyzed. First, normalcy of distribution and variance of the data will be checked. If data are distributed normally and present homoscedasticity, comparison between both groups will be performed with simple tools such as Student´s *t* test. Otherwise, non-parametric tests will be used. Differences will be considered statistically significant when *p*<0.05. All patients that return to their second visit will be included in the analysis (intention to treat analysis).

**G.-** **Proposed time table**

Each specific patient will remain formally in the study for 13 months. In the first visit all pertinent data will be collected and he/she will be informed of the study´s dynamics. A photograph of the affected eye(s) will be taken in this first visit and the patient will receive a bottle with 60 capsules that need to be taken during the following 30 days. After this period, a second visit will take place where information regarding compliance with the treatment will be registered. In addition, a second photograph of the lesion(s) will be taken. After this, surgical resection of the pterygium will be performed in those patients that need it in accordance with the protocols of the Ophthalmology Service. All procedures will be done in the same way and by a maximum of 2 ophthalmologists. Two more visits will be scheduled at 6 and 12 months after the second one. In these sessions, the evolution of the lesion will be recorded and special attention will be paid to potential recurrences, in which cases the protocols of the Ophthalmology Service will be followed.

As it pertains to the whole study, 18 months seem to be enough to recruit the needed 98 patients. If we add the 13 months needed for the last patient to complete his/her follow-up, the study could close 31 months after it opens. We propose initiating the study on February 2009 and closing up on August 2011.

**H.-** **Security and ethical considerations**

Doxycycline is drug with a very high security profile and very well known.

A short-term treatment with doxycycline has very low risks since this drug has been used for decades in the clinic (Brandt et al., Effects of doxycycline on progression of osteoarthritis. Results of a randomized, placebo-controlled, double blind trial. Arthritis Rheum. 52:2015-2025; 2005). If the treatment works in a similar way to what was observed in mice, patients could benefit from a reduction in their pterygium lesion, being even able to postpone their surgical intervention.

In patients receiving placebo no benefits are expected but no major damages are expected either, since the only think we ask is for their surgical intervention to be delayed for 4 weeks. In this period the expected growth of the lesion is minimal.

No vulnerable populations will be included.

No economic compensations are contemplated.

All the information that will be given to the patients, their family, and legal representatives is collected in the Information to Patients Document.

No additional explorations will be needed. The habitual care for pterygium patients will be applied to all participants.

All monitorizations, audits, revisions by the Local Committee for Clinical Research, and regulatory inspections related to the study will be allowed, facilitating direct access to original documents and data.

Placebo will be used as a control since no pharmacological treatment has been approved for the treatment of pterygium.
